# A multifaceted approach for analyzing complex phenotypic data in rodent models of autism

**DOI:** 10.1186/s13229-019-0263-7

**Published:** 2019-03-12

**Authors:** Ishita Das, Marcel A. Estevez, Anjali A. Sarkar, Sharmila Banerjee-Basu

**Affiliations:** MindSpec Inc., 8280 Greensboro Drive, Suite 150, McLean, VA 22102 USA

## Abstract

Autism (MIM 209850) is a multifactorial disorder with a broad clinical presentation. A number of high-confidence ASD risk genes are known; however, the contribution of non-genetic environmental factors towards ASD remains largely uncertain. Here, we present a bioinformatics resource of genetic and induced models of ASD developed using a shared annotation platform. Using this data, we depict the intricate trends in the research approaches to analyze rodent models of ASD. We identify the top 30 most frequently studied phenotypes extracted from rodent models of ASD based on 787 publications. As expected, many of these include animal model equivalents of the “core” phenotypes associated with ASD, such as impairments in social behavior and repetitive behavior, as well as several comorbid features of ASD including anxiety, seizures, and motor-control deficits. These phenotypes have also been studied in models based on a broad range of environmental inducers present in the database, of which gestational exposure to valproic acid (VPA) and maternal immune activation models comprising lipopolysaccharide (LPS) and poly I:C are the most studied. In our unique dataset of rescue models, we identify 24 pharmaceutical agents tested on established models derived from various ASD genes and CNV loci for their efficacy in mitigating symptoms relevant for ASD. As a case study, we analyze a large collection of Shank3 mouse models providing a high-resolution view of the in vivo role of this high-confidence ASD gene, which is the gateway towards understanding and dissecting the heterogeneous phenotypes seen in single-gene models of ASD. The trends described in this study could be useful for researchers to compare ASD models and to establish a complete profile for all relevant animal models in ASD research.

**Electronic supplementary material:**

The online version of this article (10.1186/s13229-019-0263-7) contains supplementary material, which is available to authorized users.

## Introduction

Animal models have been pivotal in understanding the etiology of many human diseases and determining effective therapeutic interventions [1]. Research using animal models has unearthed mechanistic underpinnings and identified therapeutic targets for neurological disorders arising due to dysfunction of specific cell types or brain regions, e.g., Parkinson’s disease [2]. Rodent models for diseases caused by viral or bacterial infections including some types of cancer and acquired immune deficiency syndrome (AIDS) have also led to an understanding of the fundamentals of the mammalian immune system leading to practical advances in healthcare management [3–5]. However, for complex behavioral disorders that have a more diffused pattern, with multiple and sporadic genomic loci implicated in disease development, rodent models have evoked more questions than answers, be it schizophrenia, Down syndrome, or ASD [6–8].

AutDB has focused on curating and annotating ASD research for the past 10 years using a scientific annotation framework rooted in the biology of the disorder [9]. The systematic annotation of autism-related data on a standardized platform has been an invaluable resource to researchers seeking to sort through confounding and groundbreaking findings. Towards this end, ASD-associated AutDB gene and copy number variant (CNV) datasets have been used widely by the research community to understand the genetic heterogeneity of ASD [10–12].

The *Animal Model* (AM) module of AutDB was created to add depth and precision to the annotation of ASD-related models. The genetic models in AM are integrated with the corresponding gene in the human gene module of AutDB, providing human genetic evidence underlying each rodent model. Our database also includes various types of environmentally induced models for autism reported in the scientific literature. Several prenatal factors, including exposure to drugs [13], role of paternal age [14], and maternal immune factors circulating during gestation [15], are being studied as causative or modulatory inducers of ASD. Additionally, the complex effects of chemical exposure and drugs after birth are also undergoing scrutiny [16–21]. In contrast to Mouse Genome Informatics (MGI), the paramount resource for mouse genetics for over 35 years, AutDB is a specialized resource that includes diverse types of ASD-related animal models, evidence or hypothesis-based, including inbred strains showing face validity to ASD, annotated using a shared and standardized framework. Another distinctive and unique feature of the AutDB resource is the inclusion of rescue models, in which drugs and procedural, genetic, or dietary manipulations are used in rodent ASD models in an attempt to rescue ASD-relevant phenotypes. Together, AutDB represents a comprehensive resource including genetic and non-genetic animal models relevant in ASD biology.

Using data curated in the AM module over the past 8 years, we demonstrate characteristic patterns in analyses undertaken to study genetic and environmentally induced rodent models of ASD. Analysis of trends based on rodent model findings shows that the most frequently assessed phenotypes are related to core features of human ASD such as social interactions, ultrasonic vocalization, and repetitive behavior. Additionally, neuroanatomical features like changes in dendritic architecture, observed in postmortem human studies of ASD brains [22], are also frequently examined in rodent models along with electrophysiology conducted on acute brain slices. These phenotypes could serve as a baseline in comparative studies of ASD models as a way of depicting complex behavioral phenotypes, related to underlying neurological substrates. With a view to facilitating translational research, we highlight pharmaceutical drugs administered to several ASD models. Finally, as a case study, we present a comprehensive analysis of phenotypes studied in rodent models of Shank3, one of the leading genetic risk factors of ASD.

## Results

### Representation of animal models in AutDB

**Fig. 1:**
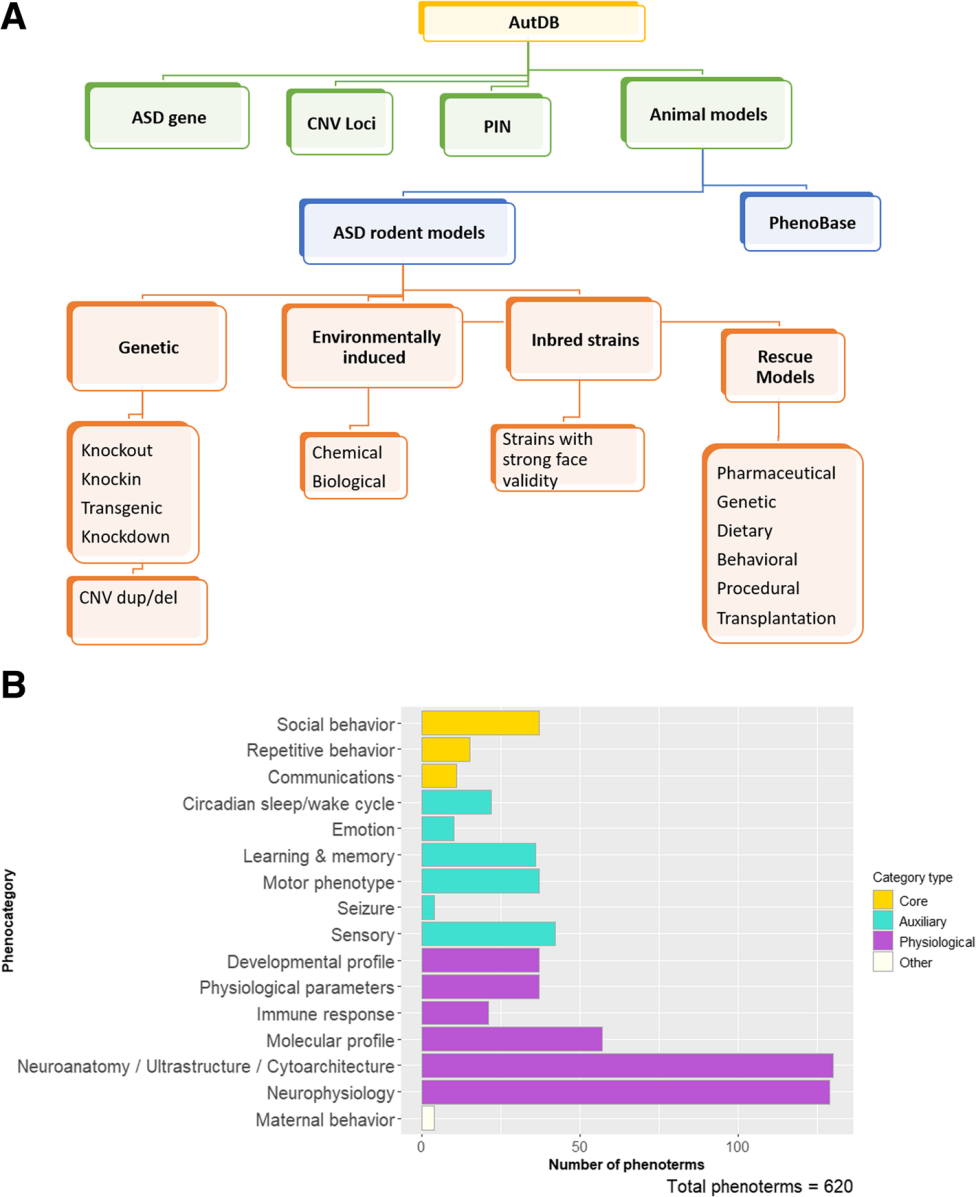
Curation of animal models in AutDB. **a** AutDB features a modular framework that aims at collating the multifactorial risk architecture associated with ASD: (1) Human Gene module curates all known human genes linked to ASD together with the detailed description of variants associated with the disorder; (2) copy number variant (CNV) module catalogs deletions and duplications of chromosomal loci implicated in ASD; (3) protein interactions (PIN) module builds networks of interacting proteins implicated in the etiology of ASD; and (4) Animal Model (AM) module collects behavioral, anatomical, and physiological data corresponding to genetic and induced models of ASD. A multilevel data-integration strategy is used to connect animal models to the corresponding entries in the Human Gene and CNV modules, respectively. Inclusion of models originating from relevant non-genetic risk factors and potential quantitative trait loci expands the repertoire of ASD models. Uniquely, AM includes rescue lines based on ASD models which have been treated with an agent to alleviate ASD-related symptoms. **b** The number of phenoterms in the Phenobase by category (phenocategory). The categories are qualified as “core” and “auxiliary”, depending on the extent of relation (accepted by the animal model research community) of the phenotypes (phenoterms) to core or auxiliary endophenotypes observed in human ASD, whereas “physiological” and “other” refer to categories that are assessed in rodents frequently but has no definitive parallels to human ASD

AutDB is an open-access portal designed to provide a comprehensive view of risk factors associated with ASD. Adopting a systems biology approach, this resource integrates diverse functional information of ASD risk factors while conserving their biological relationships. The AM module develops on the *Human Gene* and *CNV* modules of AutDB by including detailed phenotypic information of animal models. All genetic models are connected to the corresponding factor curated in AutDB for their relevance to ASD (Fig. 1). The AM module catalogs induced models based on environmental risk factors and inbred strains with ASD-consistent phenotypes. Information regarding animal models is extracted from published, peer-reviewed primary reports and parsed to provide a detailed view of the constructs and corresponding phenotypic data. The annotation of animal models is guided by a metadata repository of phenotypic terms (phenoterms) and experimental paradigms known as Phenobase [23]. Phenoterms are systematically classified into 16 broad categories that align with human ASD phenotypic features (Fig. 1b, Additional file 1: Table S1). Finally, AutDB integrates data from both mouse and rat models using a shared annotation framework for robust analysis.

### Overview of data

**Fig. 2:**
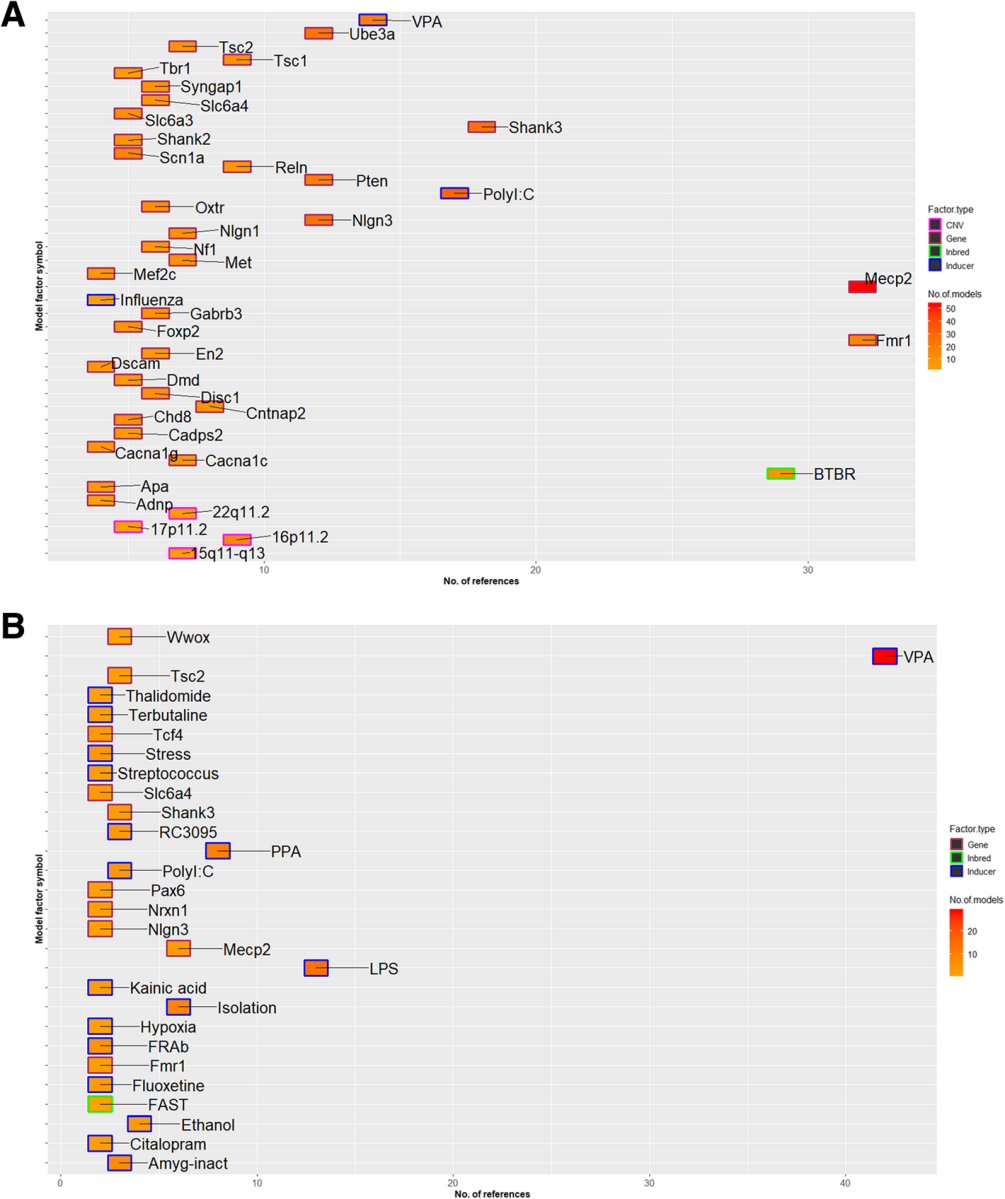
Representation of ASD-associated factors in the rodent datasets of AutDB. **a** The number of references and models annotated by the type of ASD factor in the mouse dataset. **b** The number of references and models by the type of ASD factor in the rat dataset. The type of the ASD-associated factors is shown by distinct outline colors: gene (red), CNV (pink), induced (blue), and inbred (green). Note that only factors that have four or more references are shown in the mouse (**a**) and two or more references are shown in rat (**b**). VPA, valproic acid; Poly I:C, polyinosinic:polycytidylic acid; APA, advanced paternal age; PPA, propionic acid; LPS, lipopolysaccharide; FRAb, folate receptor antibody; FAST, seizure-prone rat

The ASD rodent models described in this study were based on over 258 genes, 6 CNV regions, 72 inducers, and 9 inbred strains. These are referred to as “ASD factors” henceforth. Initial analysis indicated that the mouse models were predominantly based on genetic factors, while rat models mainly comprised environmental inducers (Additional file 2: Figure S1). A wide variation in the number of publications per ASD factor was also observed (Fig. 2a). The most annotated ASD factors (> 20 articles per factor) included the syndromic genes (Mecp2 and Fmr1) and the inbred strain (BTBR T+ Itpr3tf/J (BTBR)) in mouse (Fig. 2a) and valproic acid (VPA) in rat (Fig. 2b). ASD models based on face validity such as inbred strain BTBR were among the highly annotated factors reflecting an intense focus of the research community. The next tier of annotation included high-confidence *ASD* genes (Shank3, Chd8, Pten, Adnp), syndromic genes (Tsc1, Tsc2, Ube3a), recurrent CNVs (16p11.2, 22q11.2), and non-genetic factors (polyinosinic:polycytidylic acid (poly I:C) and VPA in mouse; lipopolysaccharide (LPS) in rat). Increasing evidence for non-genetic factors linked to ASD etiology, such as maternal immune activation (MIA), has propelled the development of such rodent models. Several mouse and rat models based on MIA induction by agents such as the influenza virus; poly I:C, a viral mimetic; or LPS, a bacterial mimetic, are represented prominently in the rodent dataset. Models based on inducing chronic or acute inflammation in pregnant dams by exposure to stress or high-fat diet or in neonates by exposure to hypoxia or isolation, respectively, have also been developed in both mice and rat. Finally, a number of environmental factors with uncertain links to ASD such as citalopram, thalidomide, terbutaline, kainic acid, stress, and maternal isolation also comprise the rat dataset (Fig. 2b).

### Signature data from ASD rodent models

**Fig. 3:**
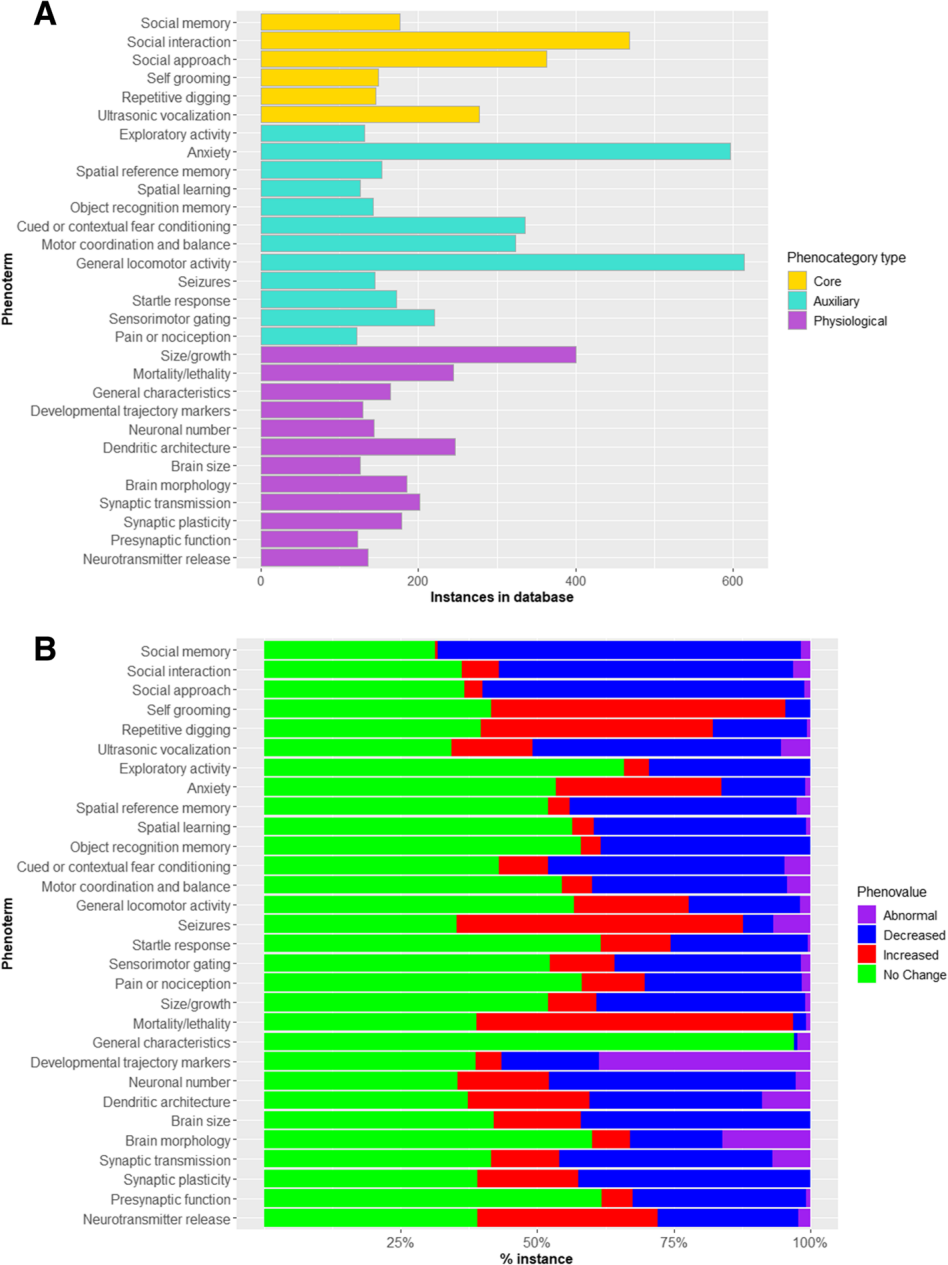
Signature data in AutDB. **a** Characteristics of the top 30 phenotypic terms in the rodent dataset. The number of instances of the most frequently annotated phenotypic terms is organized in their respective categories. The categories are color-coded indicating core phenotypes (ochre), associated phenotypes (green), or physiological observations (purple). **b** Changes observed in the most frequently annotated phenotypic terms in rodent datasets. The percentage of total instances of different valences for phenoterms are color-coded: abnormal (purple), decreased (blue), increased (red), and no change (green). Note that the phenocategory “Molecular Profile” is not included in this figure

To explore the data annotated in AutDB, which represents a comprehensive segment of research into ASD etiology, we analyzed the patterns in observations made in rodent models. In all, the data represents phenotypes of rodent models based on 345 ASD factors taken from 787 references. The frequency of phenoterm use across models is a measure of the validity of the phenoterm as a node of comparison between ASD models. We determined the top 30 most frequently annotated phenoterms in AutDB are distributed across core, auxiliary, and physiological categories (Fig. 3a). Specifically, we find observed changes in “General locomotor activity,” “Anxiety,” and “Social interaction” as the three most frequent phenoterms annotated in AutDB.

In our annotation, phenoterms are paired with a qualitative value term or “phenovalue” to indicate the direction of change compared to control animals. Phenovalues for ASD models can be “Increased,” “Decreased,” “Abnormal,” or “No Change.” This is a key feature for building the phenotypic profile of ASD models reported in hundreds of scientific reports. We mapped the percentage distribution of phenovalues for the 30 most frequently used phenoterms (Fig. 3b). Phenoterms in core categories showed higher incidence of ASD-consistent measures of phenovalue: “Ultrasonic vocalization” (Decreased= 45%; Increased = 15%), “Social memory” (Decreased= 66%), “Social interaction” (Decreased= 54%), “Social approach” (Decreased= 59%), “Self-grooming” (Increased= 54%), and “Repetitive digging” (Decreased= 17%; Increased= 42%). However, in 14 out of the 30 phenoterms, “No Change” accounted for more than 50% of the annotation for that phenoterm. These mostly represent standard control measures conducted in disease models to assess the validity and negate or account for confounding factors that can affect complex behavioral tasks. For example, animals may be tested for “Startle response” prior to testing “Cued or contextual fear conditioning” to ascertain that normal freezing response is preserved in the disease model being tested. Some of the most frequently used phenoterms from auxiliary categories are also routinely tested in various disease models of neurodevelopmental disorders, like “Spatial learning.” The phenoterms from physiological categories are frequently tested in rodent ASD models with variable outcomes. Interestingly, the phenoterms “Synaptic plasticity” and “Synaptic transmission” are reported as “Decreased” 39% and 42% of the time, respectively, reflecting a heterogeneous contribution of ASD factors towards synaptic function.

### Rescue models

In AutDB, rescue models originate from established ASD models (genetic, induced, or inbred) undergoing a treatment protocol with the aim of alleviating one or more ASD-related symptoms. A rescue paradigm is defined based on a unique combination of rescue agent, dosage, and timing of treatment. Rescue agents are further categorized according to the type of intervention (Additional file 1: Table S2). In some cases, rescue models provide an understanding of mechanisms underlying ASD-related phenotypes whereas, in other studies, rescue models employ pharmaceutical agents (e.g., FDA-approved memantine and rapamycin) to establish or validate their therapeutic use ASD patients.

**Fig. 4:**
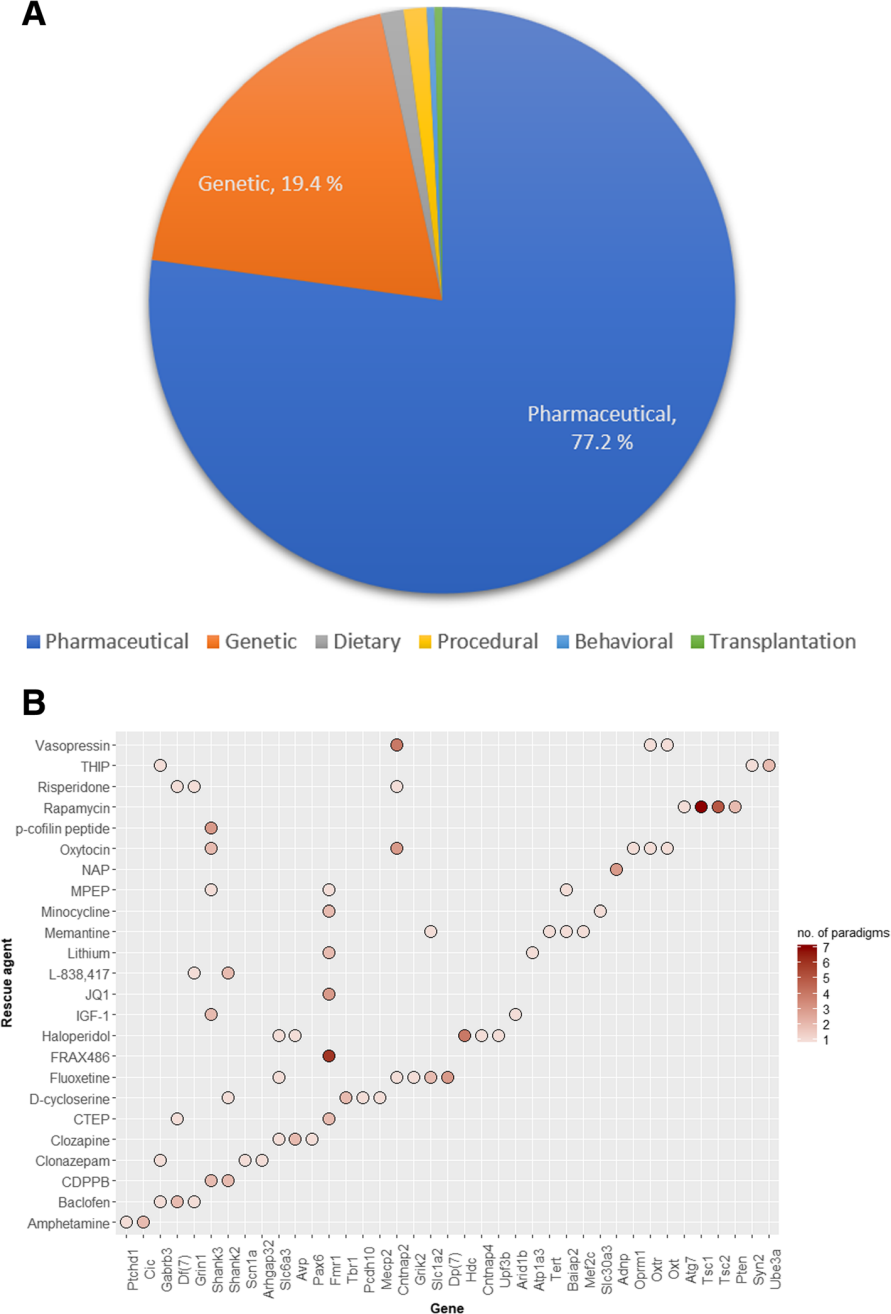
Pharmaceutical agents used in alleviating ASD-related symptoms in rodent models. **a** The pie chart shows the percentage of rescue paradigms based on different types of interventions: pharmaceutical agents, genetic, dietary, or procedural tested on genetic rodent models of ASD. **b** This scatter plot depicts the pharmaceutical agents tested on rodent ASD models based on the genes shown in the *X* axis. *Y* axis indicates name of the rescue agent. Only agents c rescue paradigm are shown. The fill gradient represents the "number of paradigms", referring to the number of treatment paradigms tested on models based on the same gene

As noted before, genetic models comprise the most frequently annotated entities in AutDB (Fig. 2). Additionally, the majority of rescue paradigms tested on genetic models of ASD are based on pharmaceutical interventions (Fig. 4a). Therefore, we focused on this dataset comprising a total of 123 pharmaceutical agents that were identified in various rescue paradigms in AutDB. We further identified 24 drugs used in more than 1 paradigm indicating the prevalent credence in their putative role in ASD therapy (Fig. 4b). Notably, in the list of 32 genes obtained using this prioritization, 10 are key ASD genes with multiple lines of human genetic evidence (SFARI categories 1–2), 3 are well-known syndromic genes, 8 are genes with suggestive evidence (category 3), and 11 are lower scoring or functional genes where the link to ASD is not fully established from human genetic evidence (Additional file 1: Table S3). The correlation between the pharmaceutical rescue agents and the targeted ASD rodent models are shown in Fig. 4b. Given the importance of altered E:I ratio in ASD [24], several drugs based on the common molecular substrate of glutamate (*N*-methyl-d-aspartate (NMDA)) or metabotropic (mGluR) receptors were tested on multiple genetic models of ASD (Table 1). Interestingly, three different drugs functioning as agonists for gamma-aminobutyric acid receptor subunit A (GABA-a) rescued ASD-related symptoms in multiple models arising from important ASD genes (SHANK2, SCN1A, GABRB3, GRIN1, UBE3A, ARHGAP32). Some of the other frequently tested drugs are currently FDA approved for the treatment of psychiatric disorders including schizophrenia (risperidone, haloperidol, clozapine), attention deficit/hyperactivity disorder (amphetamine), Alzheimer’s (memantine), bipolar disorder (lithium, risperidone), and depression (fluoxetine). Table 1 enlists the ten drugs that are currently in clinical trials for ASD. A small subset of drugs was only tested on models based on a single gene: JQ1 and FRAX48 in Fmr1 models, NAP in Adnp models, and p-cofilin in Shank3 models. In these cases, the drugs targeted specific pathways regulated by the ASD gene (Additional file 1: Table S4). For example, JQ1 is an inhibitor of bromo- and extraterminal domain (BET) proteins that are regulated by fragile X mental retardation protein (FMRP). Our database is updated quarterly with new reports that include studies corroborating the ameliorating effects of existing rescue drugs, testing overlapping and new subsets of ASD-related phenotypes.

**Table 1:** Target and/or mechanism of drugs tested on genetic rodent models of ASD which are FDA approved or in clinical trials for any clinical disorder

Agent	Target/mechanism	References	Clinical trials in ASD
IGF-1	Agonist: receptor kinase(tyrosine), Akt, IGF-1(receptor), insulin (receptor)	[40]	Yes, 2
Amphetamine	Agonist: dopamine(receptor), norepinephrine(receptor), glutamate (ionotropic receptor NMDA), serotonin(receptor)	[41]	Yes, 1 (with ADHD)
Minocycline	Neuroprotective, antibacterial and anti-inflammatory agent; inhibits: Tnf-alpha, nitric oxide synthase	[42]	Yes, 1
D-cycloserine	Agonist: glutamate (ionotropic receptor NMDA)	[43]	Yes, 1
Vasopressin	Agonist or upregulation: avp (receptor 1a/1b/2), autophagy, PIP pathway	[44]	Yes, ~ 3
Memantine	Antagonist: glutamate (ionotropic receptor NMDA), serotonin (receptor 5-HT3), acetylcholine (nicotinic receptor); agonist: sigmaergic (receptor 1), dopamine (receptor D2)	[45]	Yes, ~ 10
Baclofen	Agonist: gaba (receptor B)	[46]	Yes, ~ 8 (arbaclofen)
Risperidone	Antagonist: dopamine (receptor D1/D5/D2/D3/D4), serotonin(receptors(5-HT2a/2c), adrenergic (receptor alpha1/alpha2), histamine (receptor H1)	[47]	Yes, ~ 7
Oxytocin	Agonist: oxytocin(receptor)	[48]	Yes, ~ 24

Clinical trial information was obtained from https://clinicaltrials.gov/

### Case study: Shank3

**Fig. 5:**
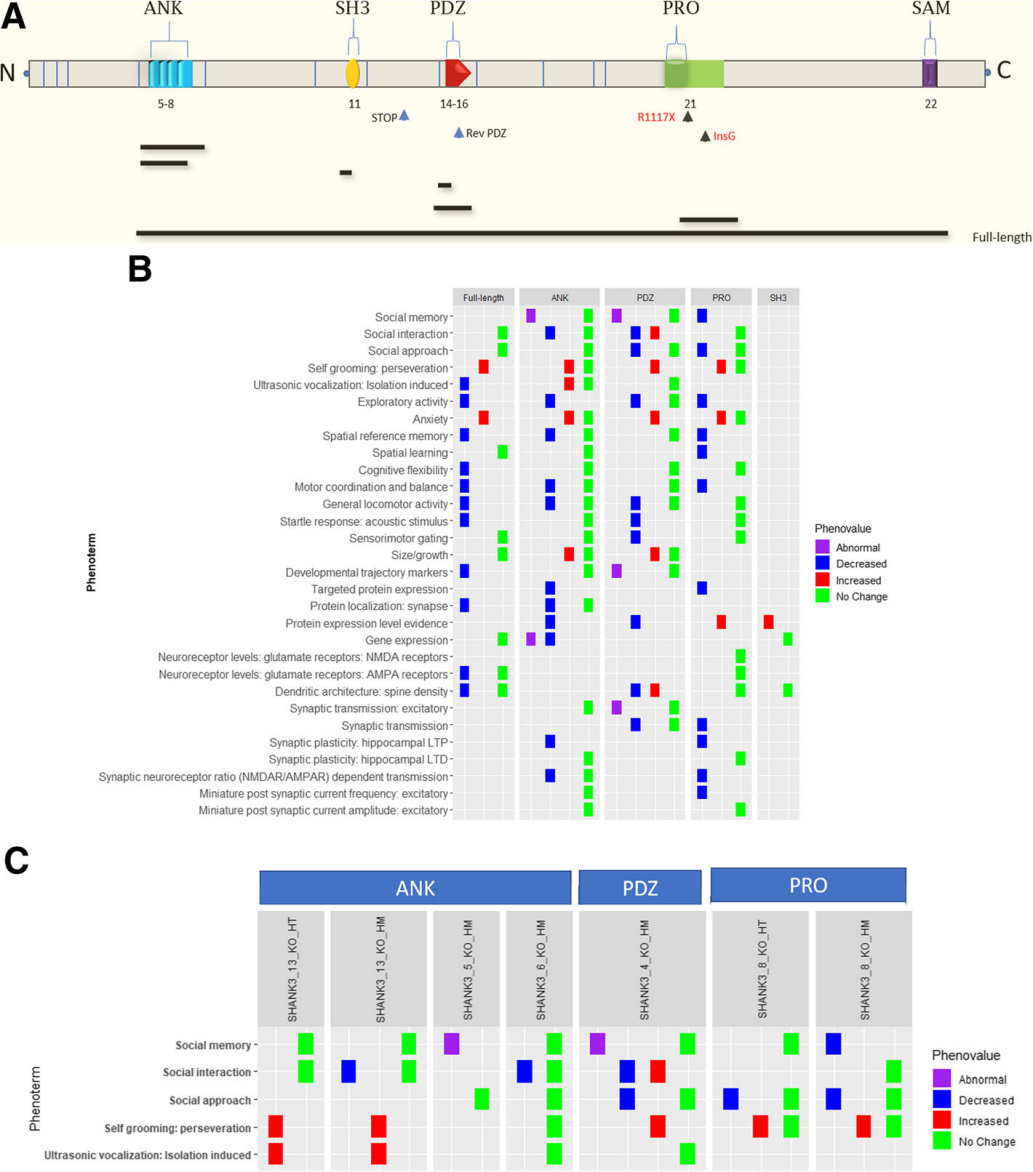
Shank3 model characteristics displayed by domain targeted. **a** Schematic representation of the relative positions of exons (blue vertical lines) and protein domains in mouse Shank3, with numbers marking exons containing the respective domains (indicated by brackets on top). The solid horizontal black lines indicate the targeted regions in Shank3 KO mouse model constructs present in AutDB. The arrow indicates the approximate location of KI constructs, and black arrows are human mutations replicated in mouse. Relative positions of introns and exons are based on Ensembl. **b** This graphic depicts the most frequently assessed phenotypes of Shank3 mouse models present in our database, displayed here separately for all homozygous (HM) KOs genotypes. Each tile color represents the phenovalue and phenotypes that have been arranged in a grid by the domain targeted in the KO constructs. **c** Detailed view of core phenotypes of Shank3 HT and HM models based on ANK-, PDZ-, and PRO-targeted domains. This figure depicts that several core phenotypes are exhibited by HT and HM models and shows the presence of apparently contradictory data in behavioral analyses, which in some instances can be explained due to differences in age or sex of animals tested (see the main text)

While studying the overall phenotypes in a large set of ASD models provided an overview of ASD literature and trends in research, an in-depth analysis of an important ASD-linked genetic factor, Shank3, illustrates the functionality of the intricate annotation in AutDB. Shank3, a multi-domain scaffolding protein with a prominent role in glutamatergic synapses, has been implicated in ASD through the identification of rare damaging mutations in multiple studies (high-confidence SFARI Gene). Accordingly, several research groups have reported mouse models of Shank3 in an attempt to define its contributory role in ASD. However, the complex structure of the Shank3 gene with multiple exons and experimentally validated alternative splicing from intragenic promoters presented a major challenge in establishing relevant animal models. The regions targeted in mouse models that are annotated in AutDB are shown in Fig. 5a. Overall, there are 27 loss-of-function (LOF) models of Shank3 including 15 knockout (KO) and 10 knockin (KI) mouse models together with 2 KO rat models (see Additional file 1: Table S8 and Additional file 3: Table S9 for details on domains targeted and model constructs). Targeting these protein domains in Shank3 resulted in different sets of isoforms being lost, further adding to the resultant phenotypic complexity [25, 26].

**Fig. 6:**
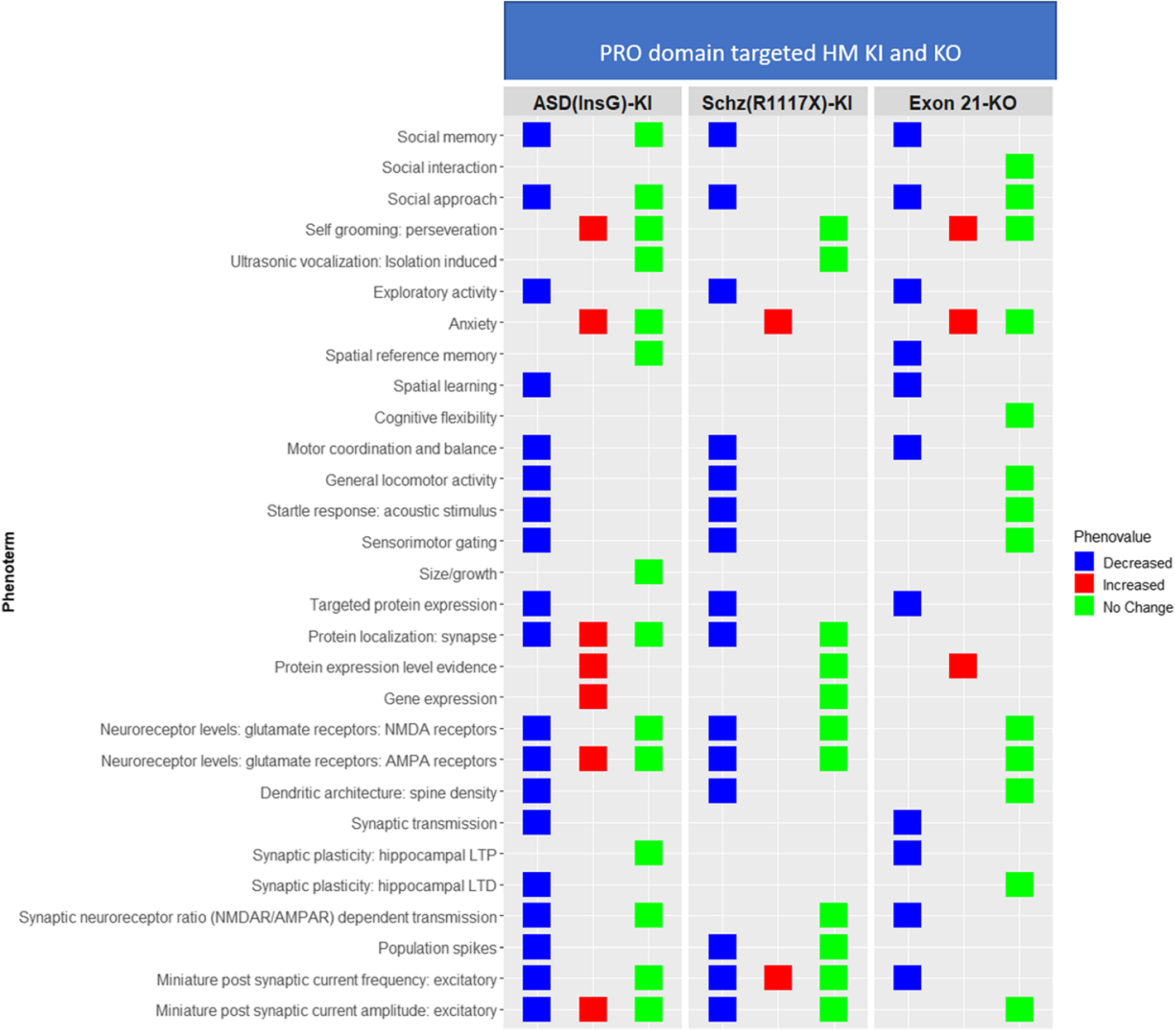
Shank3 PRO domain-targeted HM KI and KO models. Phenotypic observations from Shank3 HM KI models by insertion type. ASD(insG) and Schz(R1117X) are human mutations in exon 21 found in people with ASD and schizophrenia. Social behavior is affected in ASD and Schz-related mutations, as well as exon 21 KO that causes the production of fewer Shank3 isoforms. Schz-related mutation does not cause an increase in self-grooming, unlike the InsG, including ASD-related InsG. Other learning and memory phenotypes and neurophysiological and anatomical features are affected in both ASD and Schz-specific KI mutation as well as exon 21 KOs

Unlike many ASD genes essential for survival [27], homozygous (HM) loss of Shank3 is compatible with life through developmental stages to adulthood in both mouse and rat. Consequently, a broad range of assays targeting ASD-related core and associated phenotypes were performed in various Shank3 models (Additional file 4: Figure S2). The most frequently assessed phenotypes in Shank3 mouse models were similar to the observed trends in the entire set of ASD models in AutDB (Additional file 1: Table S5). Both “Anxiety” and “Self-grooming” were increased in all tested HM KO mouse models (Fig. 5b) indicating a strong recapitulation of human ASD phenotypes in Shank3 KOs. Similar in essence to the allelic heterogeneity in human ASD phenotypes [28], a wide variability of phenotypes in Shank3 KO models was observed based on the targeted domain. Surprisingly, full-length KOs displayed normal social behavior, whereas some deficits in social behavior were seen in models targeting ANK, PDZ, and PRO regions. Ultrasonic vocalization (USV), another “core” phenotype postulated to assay for communicative behavior, was also variably affected in the different Shank3 models, with full-length and ANK-targeted models displaying impairments in USV calls while models with mutated PDZ domains showed normal USV phenotype. Dendritic spine density in the brain, another aberrant phenotype in ASD patients, was found to be anomalous in all HM Shank3 KO mutants (Fig. 5b; AutDB). An expanded view of the top five “core” phenoterms indicates that not all phenotypes have been tested in individual constructs. Interestingly, Shank3 heterozygous (HT) mutants manifest some ASD phenotypes indicating that the dosage and nature of knocked-out Shank3 isoforms are essential for normal function (Additional file 5: Figure S3, Additional file 6: Figure S4, and Additional file 7: Figure S5). Two human mutations in exon 21 have also been recreated in Shank3 KI models, the ASD-related InsG and the Schizophrenia (Schz)-related R1117X [29, 30]. As shown in Fig. 6, compared to exon 21 KO mutants, the KI mutants have similar changes in anxiety and the InsG KI mutants also show similar changes in synaptic transmission to the KOs. Observations from rat models of Shank3, recently added to the AM module, indicate that rats and mice display differential changes in behavioral phenotypes (Additional file 8: Figure S6).

While several rescue paradigms have been tested on Shank3 models, they were all based on eight LOF models. A genetic approach based on the reinstatement of wildtype Shank3 expression after birth resulted in the alleviation of a subset of ASD phenotypes (Additional file 1: Table S6). Pharmaceutical agents like MPEP, TG003, p-cofilin, and CDPPB (3-cyano-*N*-(1,3-diphenyl-1H-pyrazol-5-yl)benzamide) (Additional file 1: Table S4) have been used in different Shank3 models (Table 2). High doses of p-cofilin, TG003, and MPEP restored or ameliorated repetitive self-grooming seen in the Shank3 mutants. Interestingly, p-cofilin and TG003 also normalize social behavior with a sustained effect (Additional file 1: Table S7). Rescue attempts on rat Shank3 models have, in turn, utilized oxytocin, which restored some deficits in social behavior and reward reinforced choice behavior (Table 3). Furthermore, neuroreceptor activity and synaptic neuroreceptor-based transmission were restored in ANK and PRO domain-targeted Shank3 mutants treated with p-cofilin and IGF-1. In the rat Shank3 model, synaptic plasticity is found to be completely restored in the medial prefrontal cortex and partially restored in the hippocampus following intracranial injection of oxytocin, indicating an avenue for translational studies.

**Table 2:** Restored or ameliorated phenotypes in Shank3 mouse models displaying the targeted domain(s), the experimental paradigms, and the drug name

Domain	Phenoterm	Experimental paradigm	Effect	Agent	Citation
All	Reward reinforced choice behavior	Operant conditioning paradigm	Ameliorated	CDPPB	[49]
	Protein localization: synapse	Western blot: striatum	Ameliorated		
	Synaptic plasticity: striatal LTD	Whole-cell patch clamp	Restored		
	General locomotor activity	Open field test	Restored	MPEP	
	Self-grooming: perseveration	Grooming behavior assessments	Ameliorated		
ANK	Motor coordination and balance	Accelerating rotarod test	Restored	IGF-1(high dose)	[50]
	Neuroreceptor activity	Whole-cell patch clamp	Restored		
	Synaptic plasticity: hippocampal LTP		Restored		
	Neuroreceptor activity		Restored	Peptide derivative of IGF-1	
	Synaptic plasticity: hippocampal LTP		Restored		
PRO	Synaptic transmission: excitatory	Whole-cell patch clamp	Ameliorated	Constitutively active Rac1	[51]
	Social approach	Three-chamber social approach test	Ameliorated		
	Cytoskeletal organization	Western blot: actin and F-actin levels	Restored	P-cofilin peptide (high dose)	
	Self-grooming: perseveration	Grooming behavior assessments	Restored		
	Synaptic neuroreceptor ratio (NMDAR/AMPAR) dependent transmission	Whole-cell patch clamp	Restored	P-cofilin peptide (high dose)^#^	
	Synaptic transmission: excitatory		Restored		
	Social approach	Three-chamber social approach test	Restored		
	Protein phosphorylation	Western blot	Restored	TG003, CLK2 inhibitor	[52]
	Self-grooming: perseveration	Grooming behavior assessments	Ameliorated		
	Social approach	Three-chamber social approach test	Restored		

^#^P-cofilin delivered by intravenous injection or stereotaxic injection into the prefrontal cortex, administered to different groups of mice, and tested separately

**Table 3:** Restored or ameliorated phenotypes in Shank3 rat models displaying the targeted domain, the experimental paradigms and drug name

Domain	Phenoterm	Experimental paradigm	Effect	Agent	Citation
ANK	Reward reinforced choice behavior	Operant conditioning paradigm	Ameliorated	Oxytocin	[53]
	Social memory: long-term social memory	Reciprocal social interaction test	Ameliorated		
	Synaptic plasticity: hippocampal LTP	Field potential recordings	Ameliorated		
	Synaptic plasticity: mPFC LTP	In vivo local field potential (LFP) recordings	Restored		

## Discussion

AutDB is a platform designed to be a specific resource where the AM module focuses on the in-depth annotation of genetic and non-genetic ASD models, using multiple layers of standardized vocabulary encapsulated in the Phenobase. Therefore, our database encompasses models based on high-confidence ASD genes (e.g., Chd8, Shank3), environmental inducers (e.g., VPA, MIA via exposure to viruses or viral mimetics like polyI:C), and inbred strains (e.g., BTBR). Rat models of ASD were recently added to AutDB to exploit interspecies conserved biology in investigating genetic and environmental ASD risk factors, also believed to be the best approach in the success of clinical trials. Additionally, rats are used in more studies assessing the effects of inducers, thereby increasing our repertoire of inducers tested in rodent ASD models. As the exact genetic signature of ASDs still remains to be determined and is a field of intense ongoing research, we believe that including putative and established models that represent characteristics of autism will lead to a more comprehensive understanding of this complex disorder.

At the time of data freeze for this article (March 2018), AutDB included rodent models based on 258 genes linked to ASD, linked to our *Human Gene* module, providing details of all rare and common variants in these genes identified in affected individuals. In contrast, mouse models based on only around 60 genes have been linked to ASD in MGI (August 2018). In addition to providing ASD-specific genetic relevance to the animal models, a number of features distinguish our annotation model from MGI. First, our integrative approach includes genetic and non-genetic models of ASD within a single platform. Second, a standardized phenotypic repository (Phenobase) structured according to the diagnostic symptoms of ASD guides the annotation of all animal models in AutDB. Third, we make dedicated provisions for all the confounding details that can give rise to contradictory data and uphold robust findings. AutDB provides distinct information on the experimental paradigms used to assess a phenotype and relevant experimental details specific to studies, information that is not available in MGI. One of the primary confounders is the experimental paradigm used to assess a phenotype, e.g., anxiety can be assessed in as many as five to six direct testing protocols, including open-field test, light-dark exploration, elevated plus maze, and novelty-induced hypophagia [31], while being reported as auxiliary observations from several other protocols like Morris water maze and social behavior testing. [32, 33]. As noted in our results, anxiety is one of the most frequently assessed phenotypes in ASD models as well. Additionally, deficiency in olfaction, vision, or perception of pain also leads to confounding measures as these underlie the sensorimotor processes necessary to complete or perform most social or learning behavioral tasks. Olfaction and pain or nociception are the most frequently annotated auxiliary measures in our database, with 116 and 122 entries by the freeze date, still among the 35 most frequent phenoterms. Olfaction is observed to be normal in over 90% cases, whereas pain or nociception has been found to be normal in 68%, with clear instances of increased or decreased perception usually taken into account for behavioral data interpretation by authors. For researchers, AutDB provides the opportunity to compare these observations and many other sensory phenotypes in all the models based on particular genes, whether or not they have been tested in every study. Our dedicated annotation of phenotypes that are indicated as “No Change” allows researchers in bioinformatics and wet labs to eliminate known confounding sources of possible multifactorial phenotypes.

In our unique rescue model dataset within the AM, we highlight test outcomes from experimental drugs, FDA-approved drugs, behavioral interventions, and transplantations of remedial cells or microbes on rodent models of ASD. We also curate genetic manipulations that have been used to rescue ASD-related phenotypes. Genetic-, behavioral-, or transplantation-based rescue paradigms play an important role in understanding the contribution of different factors for the development of phenotypes in ASD, even if not all of them can be translated into human interventions. We aim to curate new technologies as their use in ASD research is increasing; however, caveats in their application emerge retrospectively, e.g., clozapine-N-oxide (CNO) used to activate designer receptors exclusively activated by designer drugs (DREADDs) is reverse metabolized clozapine (interestingly a drug tested in ASD models) in rodents and is not pharmacologically inert [34]. Therefore, it is of great interest to the scientific community to combine the time-tested standard tasks and sophisticated frontier technology to unveil interesting relationships between different types of behavior.

The rescue model dataset of AutDB offers a comprehensive resource for translational studies. For example, genetic rescues on Shank3 models of ASD (see Additional file 1: Table S6) indicate the crucial role of normal Shank3 expression through adulthood, indicating the importance of these Shank3 mutant lines in testing remedial approaches for Shank3 mutations during postnatal development and adulthood [35]. A faster and cost-effective route for new clinical therapy in ASD is the use of existing pharmaceutical agents or “repurposing” of drugs already approved by FDA for other conditions. As seen in Fig. 4, several known drugs have been tested in genetic models, including rapamycin, fluoxetine, clozapine, and risperidone. Rescue treatment paradigms and effects, annotated discretely for rescue models in AutDB with a new set of controlled vocabulary distinct from parent ASD models, are as important as the drugs used for rescue as dosage and length of treatment can significantly change the outcome in rodents and people. This is evident in the Shank3 rescue models where a drug, p-cofilin, tailored to act on a deficit specifically manifested in Shank3 mutants; dendritic spine formation displayed differences in outcome in low (0.15 picomol/g) versus high (15 picomol/g) doses. Some of the drugs tested in Shank3 models have also been tested in other mouse models of ASD; CDPPB has been used in rescue paradigms to treat Shank2 mutants leading to the normalization of social interaction [36].

Translation from preclinical to clinical studies requires rigorous testing of a large number of paradigms, with changes in dosage and route of administration requiring. While not reported as often as favorable effects, the presence of adverse effects of drug treatments is noted in AutDB and could play an important role towards clinical drug trials. We believe refractory phenotypes should be reported whenever testing is conducted, so that the community can benefit from the knowledge and reduce unnecessary waste of resources. From the Shank3 dataset itself, it is surprising that the assessments for perseverative self-grooming and social approach or interaction were not reported after several treatments (Table 1). Overcoming this “positive data bias” is the basis for our comprehensive annotation of the “No Change” phenotype which indicates the absence of ASD-related phenotype or no statistical difference from control measures. A single model for ASD will likely not emerge from rodent studies for this genetically and clinically heterogeneous disorder. Similarly, it is unlikely that there will be a single drug prescribed for ASD therapy. As presented here, a number of the most frequent ASD-related phenotypes, like social behavior and anxiety, are assessed in Shank3 models. Additionally, based on the scaffolding function of the Shank3 at the synapse, other phenotypes like synaptic plasticity, glutamate neuroreceptor levels, and more detailed neurophysiology are also reported. This is where the advantage of rodent models lies in deciphering the constellation of phenotypes that group together and that has been the driving force for conducting the trend analyses presented here. For example, a recent study using optogenetics indicates that spatial learning can have direct effects on social behavior [37] and it is likely that future research from different fields will enhance the existing knowledge on ASD biology with new insights. Our database is primed to curate new types of paradigms and knit together cumulative observations with new discoveries. Distinctive patterns are yet to be found in ASD research; however, it is our aim to provide the platform that facilitates their detection by standardized, unbiased annotation of observations extracted from ASD scientific literature. 

## Materials and methods

### Data curation and annotation

AutDB consists of manually curated and annotated data from published, peer-reviewed scientific literature on the basis of relevance to ASD. For curation in the Animal Model module of AutDB, articles are selected for annotation based on a preliminary assessment of the validity of the rodent model and the detailed phenotypic characterization of the model. Our database is updated quarterly with annotations from the latest published literature. Details of the annotation process are documented in a wiki web resource (http://174.79.186.155:18000/AM_wiki/index.php/Rodent_annotation_guideline).

The validity of an animal model is based on its relevance to ASD. The articles must describe animal models that are based on evidence from association studies in humans, or, alternatively, models that display strong face validity for ASD-consistent endophenotypes. An animal model where an ASD-associated factor is manipulated to assess resulting phenotypes is an evidence-based model, while a model showing ASD-consistent endophenotypes for a factor with no association with ASD is a hypothesis-based model. We attempt to build a comprehensive dataset for rodent models based on high-confidence ASD genes and prioritize reports containing detailed phenotypic data on those. We only annotate differences from controls that are reported to be statistically significant (*p* value < 0.05 is the cutoff that is conventionally recorded as significant in biological studies), after careful review of figures from the main text or supplementary material. Instances where control data are either not reported or mentioned as “data not shown” are not included in AutDB annotations. Most journals have stringent policies regarding statistical testing and data analysis, so even if controlling for effect size or power for each test reported is beyond the scope of AutDB, peer review and editorial supervision are expected to take those considerations into account. Additionally, rescue models are annotated based on rescue paradigms where an agent or intervention is used to alleviate a phenotype in an ASD animal model. We categorize these rescue paradigms based on the type of agent: transplantation-based, procedural, behavioral, genetic, or pharmaceutical. We have developed annotation methods to clearly represent the treatment effects on ASD phenotypes paralleled in rodent models.

The data freeze date for all data shown in this article is March 31, 2018.

### Phenobase: dynamic hierarchical phenotypic metadata

The individual phenotypes observed in ASD models are annotated using controlled vocabulary (“phenoterms”) and organized into 16 broad categories in a resource termed as the Phenobase (Additional file 1: Table S1).These categories are grouped as “core” where the comprising phenoterms closely parallel ASD core phenotypes, “auxiliary” when the parallel human ASD phenotypes are not core diagnostic features of ASD, or “physiological” for most other associated phenotypes that are routinely assessed to determine biological underpinnings of ASD.

Phenoterms, which are arranged in a hierarchical manner, are based on endophenotypes observed in rodent models. As many complex endophenotypes of rodent animals are being reported, we have added phenoterms that capture specificity while still rooted in a broader term, such that broad terms at the top of the hierarchy are separated by colons from specific terms. For example, the term “Morphology of the basal ganglia: Striatum: Caudoputamen” specifies the morphological changes to the caudoputamen, a part of the striatum, which in turn is a part of the basal ganglia. Our phenoterms are intended to capture complex as well as simple endophenotypes that are physiological, robust or quantitative, and conserved between species. It should be noted here that the phenoterms have been developed in the context of curated ASD literature; therefore, they reflect the phenotypes assessed in ASD rodent models and not the full complexity and scope of a category per se.

In addition to phenoterms, the Phenobase contains a standardized list of experimental paradigms. This list is a discrete part of the database that is used to represent the tests used to study phenotypes in different animal models. The standardization of experimental paradigms adapts a uniform nomenclature that circumvents the variety of synonyms used in literature for similar experimental setups. The Phenobase catalogs are over 450 experimental paradigms that map to the whole set of 620 phenoterms. The combined use of phenoterm and experimental paradigm provides a more comprehensive picture of observations made by authors, which allows for better comparisons of model phenotypes between different ASD models as well as between researchers.

### Data analysis and visualization

Most data analysis and frequency measurements were conducted in R using the following packages: “tidyverse,” “dplyr,” and “forcats” [38]. Graphics were developed with ggplot2 or in excel [39].

## Additional files

Additional file 1: **Table S1.** Phenocategory definitions. **Table S2.** Rescue model type definitions. **Table S3.** SFARI scores of genes in Fig. 4b. **Table S4.** Target or mechanism of drugs tested in two or more paradigms on rodent genetic models that are not in ASD clinical trials. **Table S5.** Shank3 top 31 most frequent phenotypes including targeted protein expression. **Table S6.** Shank3 rescue data genetic reinstatement. **Table S7.** Shank3 rescue data all outcomes of drugs. **Table S8.** Shank3 domains with model IDs. (DOCX 80 kb)

Additional file 2: **Figure S1.** Distribution of ASD factor in rodent data. A. The stacked plot shows that the genes comprise the largest subtype of ASD factors in AutDB with 258 total genes present in the dataset, with most of the models developed in mice. On the other hand, inducers (72) are overrepresented by rat models and there are about equal numbers of inbred strains that show face validity to ASD in both species. The same ASD-related gene or inducer has been modeled in mice and rat infrequently, with only 19/258 genes and 8/72 inducers modeled in both. (TIF 237 kb)

Additional file 3: **Figure S2.** Shank3 model phenotypic data displayed by genotype. An overall representation of Shank3 mouse data separated only by genotype. This figure illustrates that both HM and HT Shank3 KO and KI models have been tested for many phenotypes using different constructs designs (Additional file 1: Table S8). (TIF 269 kb)

Additional file 4: **Table S9.** Shank3 model construct definitions (all KO and KI) (excel sheet). (XLSX 14 kb)

Additional file 5: **Figure S3.** Accompanying Fig. 5b. Shank3 heterozygous KO model data depicted by protein domain targeted and genotype. (TIF 167 kb)

Additional file 6: **Figure S4.** Accompanying Fig. 6. Shank3 PRO domain targeted heterozygous KI and KO model data. (TIF 273 kb)

Additional file 7: **Figure S5.** Phenotypes of Shank3 KI models. A) The HM KI models display several core phenotypes including impaired social behavior and increased self-grooming. Depending on the mutation, there is heterogeneity in the manifestation of other behavioral phenotypes like anxiety and spatial learning. The KI human mutations are discussed in main text. B) Shank3 HT KI mutant mice still manifest core phenotypes, whereas other tested behavior is more like wild-type mice, like normal anxiety, sensorimotor gating, and spatial learning. (TIF 707 kb)

Additional file 8: **Figure S6.** Rat Shank3 phenotypic data. The HT and HM rat models depicted here were developed by targeting the Ank domain. Rat models of Shank3 display some deficits in social behavior (long-term memory) but do not share several of the phenotypes displayed by mouse Shank3 models, like no impairments in ultrasonic vocalization or changes in anxiety levels are observed in rats. (TIF 105 kb)
