# Supplementary material for: A multifaceted approach for analyzing complex phenotypic data in rodent models of autism
Source: Mol Autism. 2019 Mar 12;10:11. doi: 10.1186/s13229-019-0263-7 (PMC6417187; doi:10.1186/s13229-019-0263-7)
Supplement: Supplementary file 1 — Table S1. Phenocategory definitions. Table S2. Rescue model type definitions. Table S3. SFARI scores of genes in Fig. 4b. Table S4. Target or mechanism of drugs tested in two or more paradigms on rodent genetic models that are not in ASD clinical trials. Table S5. Shank3 top 31 most frequent phenotypes including targeted protein expression. Table S6. Shank3 rescue data genetic reinstatement. Table S7. Shank3 rescue data all outcomes of drugs. Table S8. Shank3 domains with model IDs. (DOCX 80 kb) [file 13229_2019_263_MOESM1_ESM.docx]

# **Additional file 1**

**Table S1: Phenobase categories**

| **Phenocategory** | **Relevance to ASD** | **Definition** |
| --- | --- | --- |
| Social behavior | Core | Assessment of social function by behavioral testing. Includes indicators of social behavior, even in the absence of other mice or inanimate object. |
| Communication |  | Testing frequency, pattern and other properties of ultrasonic and audible vocalizations made by pups or adults |
| Repetitive behavior |  | Restricted behavior that is repeated without evidence of purpose or normal function |
| Motor phenotype | Auxiliary | Phenotypes associated with motor control of movements and reflex actions via the spinal cord and include all patterning and neuronal function characterization of peripheral neurons related to voluntary and involuntary movement. Also includes characterization of movement and coordination as the end result of neurophysiological or neuroanatomical changes. However, changes in the brain structure and function that lead to changes in the motor phenotype are NOT included in this category. |
| Sensory |  | Phenotype associated with sensory perception of external stimuli arising from touch, vision, hearing, smell (olfaction) and taste. Includes sensory organ development, neuronal patterns, organization and function, present at the level of contact with the external stimuli to the sensory neurons that convey the perception. Changes to the brain/secondary or higher order neuronal structure are not included but physiology or region of activation and its association with interpreting perception are included. |
| Learning and memory |  | Conditioned response to cues and context, memory of the training process. Includes procedural and declarative learning and memory as well as classical conditioning and Pavlovian fear response |
| Emotion |  | Includes anxiety, response to stressors like forced restraint, fear and novel environment and depression. Limited to untrained or instinctive response to the environment that does appear to incite any conditioned memory. |
| Seizure |  | Episode of abnormal electrical activity in the brain that may lead to changes in behavior or overt physiological outcome like convulsions or jerky movements. |
| Circadian sleep/wake cycle |  | Phenotypes associated with normal diurnal activity patterns of mice, behavior in light or darkness and sleep patterns. |
| Developmental Profile | Physiological | Organ system development until P21. Except in case of neuronal development patterns (embryonic lethal models) or testing that falls specifically under another category (like communications or motor phenotype). Morphology of tissue (including muscle) is included under this category. |
| Physiological parameters |  | Measurement of parameters that mark the normal functioning of organ systems and whole-body physiology, used to monitor normal body or tissue functioning |
| Immune response |  | Innate and adaptive immune response to infection or autoimmune activation indicators. Includes autoimmune activation related to oxidative stress, stroke or ischemia. Also includes all forms of inflammation and inflammatory reactions. Additionally, includes continuing development of immune cells in primary and secondary lymphoid organs in adults |
| Neurophysiology |  | All molecular, network, electrical and chemical steady state processes associated with brain function and activity. Additionally, all changes to brain activity and physiological processes in response to external stimuli including, but not limited to, learning, stress and exercising. |
| Neuroanatomy/ultrastructure/cytoarchitecture |  | Structural characterization of the brain, including cytoarchitecture, patterning of the layers, organization of specialized neurons and regions of the brain. |
| Molecular profile |  | Exploratory biochemical analyses using low or high throughput methods |
| Maternal behavior | Other | Maternal care, nurturing of preweaning pups including nest building and feeding |

Table S2. Rescue type assignments

| **Rescue types** | **Definition** |
| --- | --- |
| RESCUE-Pharmaceutical | Treatment with chemical drug or any protein/peptide/antibody that can be synthesized as a drug (in lines of insulin, antibodies) with the aim of alleviating ASD phenotypes |
| RESCUE: Dietary | Treatment with dietary components such as amino acids, fat, ketones, probiotics) etc. (please see next slide to look at distinction between part of diet and pharmaceutical) |
| RESCUE-Genetic | Genetic intervention targeted on gene expression or protein translation aimed at alleviating ASD phenotypes |
| RESCUE-Transplantation | Transplantation of bone marrow, neurons or tissue, immune cells or similar |
| RESCUE-Behavioral | Behavioral/external intervention that changes the environment of mice after birth e.g., enriched environment, increased exercise, cross fostering, cage mates or similar |
| RESCUE-Procedural | Paradigm targeting neurons or brain tissue without using specific genetic engineering (e.g., optogenetics) |

Table S3. SFARI scores of genes prioritized in figure 4b based on rescue paradigms using pharmaceutical agents.

| **Gene name** | **Gene score** | **Syndromic (Yes = 1; No =0)** |
| --- | --- | --- |
| Adnp | 1 | 1 |
| Arid1b | 1 | 1 |
| Pten | 1 | 1 |
| Shank3 | 1 | 1 |
| Tbr1 | 1 | 0 |
| Cntnap2 | 2 | 1 |
| Gabrb3 | 2 | 0 |
| Mecp2 | 2 | 1 |
| Ptchd1 | 2 | 0 |
| Shank2 | 2 | 0 |
| Cntnap4 | 3 | 0 |
| Grik2 | 3 | 0 |
| Grin1 | 3 | 0 |
| Oxtr | 3 | 0 |
| Scn1a | 3 | 1 |
| Slc6a3 | 3 | 0 |
| Tsc2 | 3 | 1 |
| Ube3a | 3 | 1 |
| Arhgap32 | 4 | 0 |
| Atp1a3 | 4 | 1 |
| Mef2c | 4 | 1 |
| Oxt | 4 | 0 |
| Pcdh10 | 4 | 0 |
| Syn2 | 4 | 0 |
| Atg7 | 5 | 0 |
| Baiap2 | 5 | 0 |
| Hdc | 5 | 0 |
| Oprm1 | 5 | 0 |
| Tert | 5 | 0 |
| Fmr1 | NA | 1 |
| Slc1a2 | NA | 1 |
| Tsc1 | NA | 1 |

Table S4. Target or mechanism of drugs tested on genetic rodent models of ASD not in any ASD clinical trial

| **Agent** | **Target/ Mechanism** | **References** |
| --- | --- | --- |
| CDPPB | agonist: glutamate (metabotropic receptor5) | (1) |
| Clonazepam | agonist: gaba (receptor A) | (2) |
| Clozapine | antagonist: serotonin (receptor 5-HT 2a/2c), dopamine (receptor) | (3) |
| CTEP | antagonist: glutamate (metabotropic receptor5) | (4) |
| Fluoxetine | antagonist: serotonin(transporter), serotonin (receptor 5HT2c) | (5) |
| FRAX486 | inhibitor: kinase(p21 activated OR Pak1) | (6) |
| Haloperidol | antagonist: dopamine (receptor D2), serotonin (receptor 5-HT2), adrenergic (receptor alpha1a, 2a,2b, 2c) | (3) |
| JQ1 | antagonist: BET proteins | (7) |
| L-838,417 | agonist(partial): gaba (receptor A) | (8) |
| Lithium | inhibitor: kinase (GSK3), PIP pathway | (9) |
| MPEP | antagonist: glutamate (metabotropic receptor5) | (10) |
| NAP (derived from activity-dependent neuroprotective protein) | neurotrophic agent, promotes axonal transport | (11) |
| p-cofilin peptide | inhibitor: microtubule(assembly) | (12) |
| Rapamycin | inhibitor: mTOR | (13) |
| THIP | agonist: gaba (receptor A) | (14) |

Table S5. Top 31 phenoterms in Shank3 mouse data

| Phenotypic.ontological.term | N |
| --- | --- |
| Anxiety | 28 |
| Protein expression level evidence | 26 |
| Miniature post synaptic current amplitude: excitatory | 23 |
| Miniature post synaptic current frequency: excitatory | 23 |
| Self grooming: perseveration | 22 |
| Social approach | 22 |
| Motor coordination and balance | 21 |
| General locomotor activity | 18 |
| Protein localization: synapse | 18 |
| Neuroreceptor levels: glutamate receptors: AMPA receptors | 15 |
| Social memory | 15 |
| Synaptic neuroreceptor ratio (NMDAR/AMPAR) dependent transmission | 15 |
| Exploratory activity | 14 |
| Neuroreceptor levels: glutamate receptors: NMDA receptors | 14 |
| Sensorimotor gating | 14 |
| Social interaction | 14 |
| Dendritic architecture: spine density | 13 |
| Startle response: acoustic stimulus | 13 |
| Size/growth | 12 |
| Spatial reference memory | 12 |
| Targeted protein expression | 12 |
| Spatial learning | 10 |
| Synaptic plasticity: hippocampal LTP | 9 |
| Synaptic transmission | 9 |
| Synaptic transmission: excitatory | 9 |
| Population spikes | 8 |
| Synaptic plasticity: hippocampal LTD | 8 |
| Ultrasonic vocalization: Isolation induced | 8 |
| Cognitive flexibility | 7 |
| Developmental trajectory markers | 7 |
| Gene expression | 7 |

Table S6. Genetically reinstating Shank3 in Shank3 mutant mice

| Domain | Phenocategory | Phenoterm | Experimental paradigm | Effect | Reinstatement of Shank3 |
| --- | --- | --- | --- | --- | --- |
| PDZ | Neuroanatomy / Ultrastructure / Cytoarchitecture | Dendritic architecture: spine density | Immunohistochemistry: AAV-GFP labeling | Restored | Adults* |
| PDZ | Neurophysiology | Miniature post synaptic current frequency: excitatory | Whole-cell patch clamp | Restored | Adults* |
| PDZ | Neurophysiology | Population spikes | Field potential recordings | Restored | Adults* |
| PDZ | Molecular profile | Protein expression level evidence | Western blot | Restored | Adults* |
| PDZ | Repetitive behavior | Self grooming: perseveration | Grooming behavior assessments | Restored | Adults* |
| PDZ | Emotion | Anxiety | Elevated zero maze test | Restored | Adults* |
| PDZ | Motor phenotype | Motor coordination and balance | Accelerating rotarod test | Restored | Adults* |
| PDZ | Social behavior | Social approach | Three-chamber social approach test | Ameliorated | Adults* |
| PDZ | Molecular profile | Targeted protein expression | Immunohistochemistry: Shank3, Cre | Ameliorated | Adults* |
| PDZ | Developmental profile | Size/growth | Body weight measurement | Side Effect | Adults* |
| PDZ | Neurophysiology | Miniature post synaptic current amplitude: excitatory | Whole-cell patch clamp | No Adverse Effect | Adults* |
| PDZ | Neuroanatomy / Ultrastructure / Cytoarchitecture | Neuroreceptor levels: glutamate receptors: NMDA receptors | Whole-cell patch clamp | No Adverse Effect | Adults* |
| PDZ | Neurophysiology | Presynaptic function: paired-pulse facilitation | Whole-cell patch clamp | No Adverse Effect | Adults* |
| PDZ | Neurophysiology | Presynaptic function: presynaptic fiber volley | Field potential recordings | No Adverse Effect | Adults* |
| PDZ | Neurophysiology | Synaptic neuroreceptor ratio (NMDAR/AMPAR) dependent transmission | Whole-cell patch clamp | No Adverse Effect | Adults* |
| PDZ | Emotion | Anxiety | Elevated zero maze test | Refractory | Adults* |
| PDZ | Motor phenotype | General locomotor activity: Ambulatory activity | Open field test | Refractory | Adults* |
| PDZ | Motor phenotype | Motor coordination and balance | Accelerating rotarod test | Refractory | Adults* |
| PDZ | Social behavior | Rearing behavior | Open field test | Refractory | Adults* |
| PDZ | Molecular profile | Targeted protein expression | Western blot: Shank3 | Ameliorated | Juveniles* |
| PDZ | Motor phenotype | General locomotor activity: Ambulatory activity | Open field test | Refractory | Juveniles* |
| PDZ | Social behavior | Rearing behavior | Open field test | Refractory | Juveniles* |
| PRO | Molecular profile | Targeted protein expression | Western blot | Restored | NA^#^ |

*Mei Y et al, Nature 2016

^#^ Speed HE et al, J. Neurosci 2015

Table S7. Other effects of drugs on various phenotypes in Shank3 mutants

| Domain | Phenocategory | Phenoterm | Effect | Experimental paradigm | Agent | Citation |
| --- | --- | --- | --- | --- | --- | --- |
| All | Neurophysiology | Action potential firing | Refractory | Whole-cell patch clamp: dorsostriatal medium spiny neurons | CDPPB | (15) |
| All | Neurophysiology | Presynaptic function | Refractory | High-frequency stimulation (HFS) | CDPPB | (15) |
| All | Repetitive behavior | Self grooming: perseveration | Side Effect | Grooming behavior assessments | CDPPB | (15) |
| All | Neurophysiology | Spontaneous post synaptic event amplitude: excitatory currents | No Adverse Effect | Whole-cell patch clamp: dorsostriatal medium spiny neurons | CDPPB | (15) |
| All | Neurophysiology | Spontaneous post synaptic event frequency: excitatory currents | Refractory | Whole-cell patch clamp: dorsostriatal medium spiny neurons | CDPPB | (15) |
| PRO | Emotion | Exploratory activity | Refractory | Marble-burying test | TG003, CLK2 inhibitor | (16) |
| PRO | Social behavior | Social approach | Sustained Effect | Three-chamber social approach test | TG003, CLK2 inhibitor | (16) |
| PRO | Emotion | Anxiety | No Adverse Effect | Open field test | p-cofilin peptide (high dose) | (12) |
| PRO | Molecular profile | Cytoskeletal organization | Sustained Effect | Western blot: actin and F-actin levels; Immunostaining: phalloidin in PFC slices | p-cofilin peptide (high dose) | (12) |
| PRO | Motor phenotype | General locomotor activity | No Adverse Effect | Open field test | p-cofilin peptide (high dose) | (12) |
| PRO | Motor phenotype | Motor coordination and balance | No Adverse Effect | Accelerating rotarod test | p-cofilin peptide (high dose) | (12) |
| PRO | Social behavior | Social approach | Sustained Effect | Three-chamber social approach test | p-cofilin peptide (high dose) | (12) |
| PRO | Neurophysiology | Synaptic neuroreceptor ratio (NMDAR/AMPAR) dependent transmission | Sustained Effect | Whole-cell patch clamp: layer V pyramidal neurons in the prefrontal cortex (evoked EPSC) | p-cofilin peptide (high dose) | (12) |
| PRO | Neurophysiology | Synaptic transmission: excitatory | Sustained Effect | Whole-cell patch clamp: layer V pyramidal neurons in the prefrontal cortex (evoked EPSC) | p-cofilin peptide (high dose) | (12) |
| PRO | Repetitive behavior | Self grooming: perseveration | Refractory | Grooming behavior assessments | p-cofilin peptide (low dose) | (12) |
| PRO | Social behavior | Social approach | Refractory | Three-chamber social approach test | p-cofilin peptide (low dose) | (12) |
| PRO | Neurophysiology | Synaptic transmission: excitatory | Refractory | Whole-cell patch clamp: layer V pyramidal neurons in the prefrontal cortex (evoked EPSC) | p-cofilin peptide (low dose) | (12) |
| PRO | Emotion | Exploratory activity | Refractory | Marble-burying test | TG003, CLK2 inhibitor | (16) |

Table S8. Shank3 model IDs with exon and domains targeted

| Model.ID | Mutation | Domain |
| --- | --- | --- |
| SHANK3_19_KO_HM | exons 4-22 | All |
| SHANK3_19_KO_HT | exons 4-22 | All |
| SHANK3_20_CKO_HM_NAc | exons 4-22 | All |
| SHANK3_19_KO_HM_CDPPB-1 | exons 4-22 | All |
| SHANK3_19_KO_HM_CDPPB-2 | exons 4-22 | All |
| SHANK3_19_KO_HM_MPEP | exons 4-22 | All |
| SHANK3_1_KO_HM | exon 4 to exon 9 | ANK |
| SHANK3_1_KO_HT | exon 4 to exon 9 | ANK |
| SHANK3_13_KO_HM | exon 4 to exon 9 | ANK |
| SHANK3_13_KO_HT | exon 4 to exon 9 | ANK |
| SHANK3_5_KO_HM | exons 4 – 7 | ANK |
| SHANK3_6_KO_HM | exons 4-9 | ANK |
| SHANK3_1_KO_HT_IGF1-H | exon 4 to exon 9 | ANK |
| SHANK3_1_KO_HT_IGF1-L | exon 4 to exon 9 | ANK |
| SHANK3_1_KO_HT_pIGF1 | exon 4 to exon 9 | ANK |
| R_SHANK3_1_KO_HT | exon 6 | ANK |
| R_SHANK3_2_KO_HM | exon 6 | ANK |
| SHANK3_23_KI_HT | intron 12-STOP | NA |
| SHANK3_23_KI_HM | intron 12-STOP | NA |
| SHANK3_21_KI_HM | Inv-PDZ | PDZ |
| SHANK3_22_KI_HM | Inv-PDZ | PDZ |
| SHANK3_4_KO_HM | exon 13 to 16' | PDZ |
| SHANK3_4_KO_HT | exon 13 to 16' | PDZ |
| SHANK3_22_KI_HM_Reinstate-Adult | Inv-PDZ | PDZ |
| SHANK3_22_KI_HM_Reinstate-Juvenile | Inv-PDZ | PDZ |
| SHANK3_10_KI_HM_InsG | ASD(InsG) | PRO |
| SHANK3_10_KI_HT_InsG | ASD(InsG) | PRO |
| SHANK3_15_KI_HM_InsG | ASD(InsG) | PRO |
| SHANK3_15_KI_HT_InsG | ASD(InsG) | PRO |
| SHANK3_17_KI_HM_R1117X | Schz(R1117X) | PRO |
| SHANK3_17_KI_HT_R1117X | Schz(R1117X) | PRO |
| SHANK3_3_KO_HT | exon 21 | PRO |
| SHANK3_8_KO_HM | exon 21 | PRO |
| SHANK3_8_KO_HT | exon 21 | PRO |
| SHANK3_10_KI_HM_InsG_Reinstate-Shank3 | exon 21 | PRO |
| SHANK3_8_KO_HT_HSV-CA-Rac1 | exon 21 | PRO |
| SHANK3_8_KO_HM_TG003 | exon 21 | PRO |
| SHANK3_8_KO_HT_TATcofilin-1 | exon 21 | PRO |
| SHANK3_8_KO_HT_TATcofilin-2 | exon 21 | PRO |
| SHANK3_8_KO_HT_TATcofilin-3 | exon 21 | PRO |
| SHANK3_8_KO_HT_TG003 | exon 21 | PRO |
| SHANK3_7_KO_HM | exon 11 | SH3 |

REFERENCES

1. Kinney GG, O'Brien JA, Lemaire W, Burno M, Bickel DJ, Clements MK, et al. A novel selective positive allosteric modulator of metabotropic glutamate receptor subtype 5 has in vivo activity and antipsychotic-like effects in rat behavioral models. J Pharmacol Exp Ther. 2005;313(1):199-206.

2. Macdonald RL, McLean MJ. Anticonvulsant drugs: mechanisms of action. Adv Neurol. 1986;44:713-36.

3. Seeman P. Atypical antipsychotics: mechanism of action. Can J Psychiatry. 2002;47(1):27-38.

4. Lindemann L, Jaeschke G, Michalon A, Vieira E, Honer M, Spooren W, et al. CTEP: a novel, potent, long-acting, and orally bioavailable metabotropic glutamate receptor 5 inhibitor. J Pharmacol Exp Ther. 2011;339(2):474-86.

5. Owens MJ, Knight DL, Nemeroff CB. Second-generation SSRIs: human monoamine transporter binding profile of escitalopram and R-fluoxetine. Biol Psychiatry. 2001;50(5):345-50.

6. Hayashi-Takagi A, Araki Y, Nakamura M, Vollrath B, Duron SG, Yan Z, et al. PAKs inhibitors ameliorate schizophrenia-associated dendritic spine deterioration in vitro and in vivo during late adolescence. Proc Natl Acad Sci U S A. 2014;111(17):6461-6.

7. Filippakopoulos P, Qi J, Picaud S, Shen Y, Smith WB, Fedorov O, et al. Selective inhibition of BET bromodomains. Nature. 2010;468(7327):1067-73.

8. Gandal MJ, Sisti J, Klook K, Ortinski PI, Leitman V, Liang Y, et al. GABAB-mediated rescue of altered excitatory-inhibitory balance, gamma synchrony and behavioral deficits following constitutive NMDAR-hypofunction. Transl Psychiatry. 2012;2:e142.

9. Jope RS. Anti-bipolar therapy: mechanism of action of lithium. Mol Psychiatry. 1999;4(2):117-28.

10. Lea PMt, Faden AI. Metabotropic glutamate receptor subtype 5 antagonists MPEP and MTEP. CNS Drug Rev. 2006;12(2):149-66.

11. Gozes I, Divinski I. NAP, a neuroprotective drug candidate in clinical trials, stimulates microtubule assembly in the living cell. Curr Alzheimer Res. 2007;4(5):507-9.

12. Duffney LJ, Zhong P, Wei J, Matas E, Cheng J, Qin L, et al. Autism-like Deficits in Shank3-Deficient Mice Are Rescued by Targeting Actin Regulators. Cell Rep. 2015;11(9):1400-13.

13. Abraham RT, Wiederrecht GJ. Immunopharmacology of rapamycin. Annu Rev Immunol. 1996;14:483-510.

14. Krogsgaard-Larsen P, Johnston GA, Lodge D, Curtis DR. A new class of GABA agonist. Nature. 1977;268(5615):53-5.

15. Wang X, Bey AL, Katz BM, Badea A, Kim N, David LK, et al. Altered mGluR5-Homer scaffolds and corticostriatal connectivity in a Shank3 complete knockout model of autism. Nat Commun. 2016;7:11459.

16. Bidinosti M, Botta P, Kruttner S, Proenca CC, Stoehr N, Bernhard M, et al. CLK2 inhibition ameliorates autistic features associated with SHANK3 deficiency. Science. 2016;351(6278):1199-203.
