# Supplementary figures and images for: A multifaceted approach for analyzing complex phenotypic data in rodent models of autism
Source: Mol Autism. 2019 Mar 12;10:11. doi: 10.1186/s13229-019-0263-7 (PMC6417187; doi:10.1186/s13229-019-0263-7)

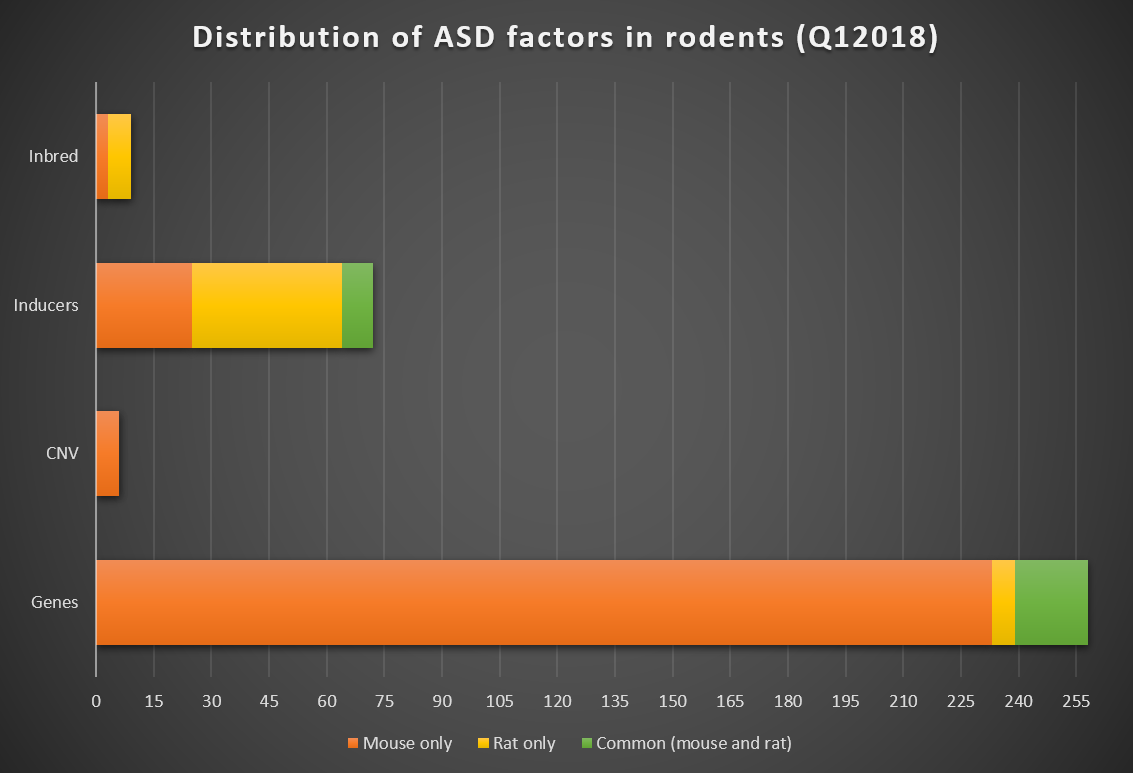

Supplement: Supplementary file 2 — Figure S1. Distribution of ASD factor in rodent data. A. The stacked plot shows that the genes comprise the largest subtype of ASD factors in AutDB with 258 total genes present in the dataset, with most of the models developed in mice. On the other hand, inducers (72) are overrepresented by rat models and there are about equal numbers of inbred strains that show face validity to ASD in both species. The same ASD-related gene or inducer has been modeled in mice and rat infrequently, with only 19/258 genes and 8/72 inducers modeled in both. (TIF 237 kb) [file 13229_2019_263_MOESM2_ESM.tif]

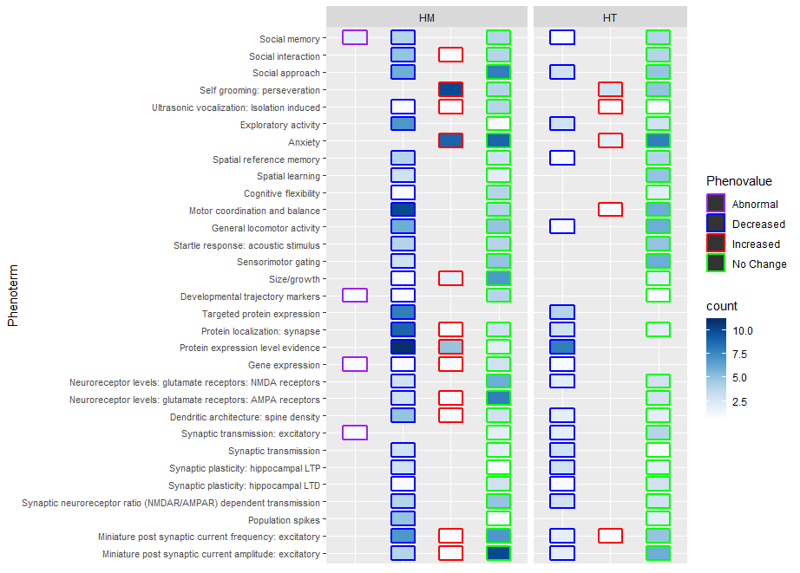

Supplement: Supplementary file 3 — Figure S2. Shank3 model phenotypic data displayed by genotype. An overall representation of Shank3 mouse data separated only by genotype. This figure illustrates that both HM and HT Shank3 KO and KI models have been tested for many phenotypes using different constructs designs (Additional file 1: Table S8). (TIF 269 kb) [file 13229_2019_263_MOESM3_ESM.tif]

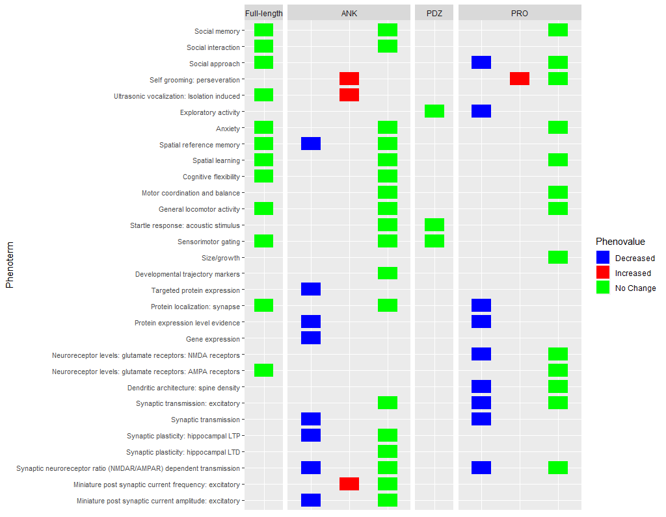

Supplement: Supplementary file 5 — Figure S3. Accompanying Fig. 5b. Shank3 heterozygous KO model data depicted by protein domain targeted and genotype. (TIF 167 kb) [file 13229_2019_263_MOESM5_ESM.tif]

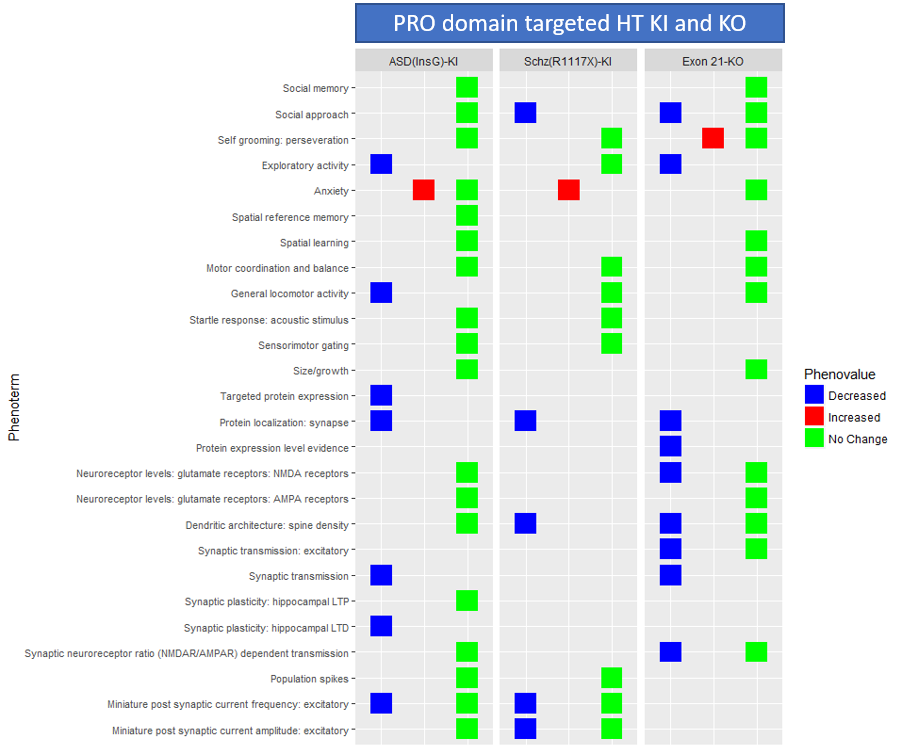

Supplement: Supplementary file 6 — Figure S4. Accompanying Fig. 6. Shank3 PRO domain targeted heterozygous KI and KO model data. (TIF 273 kb) [file 13229_2019_263_MOESM6_ESM.tif]

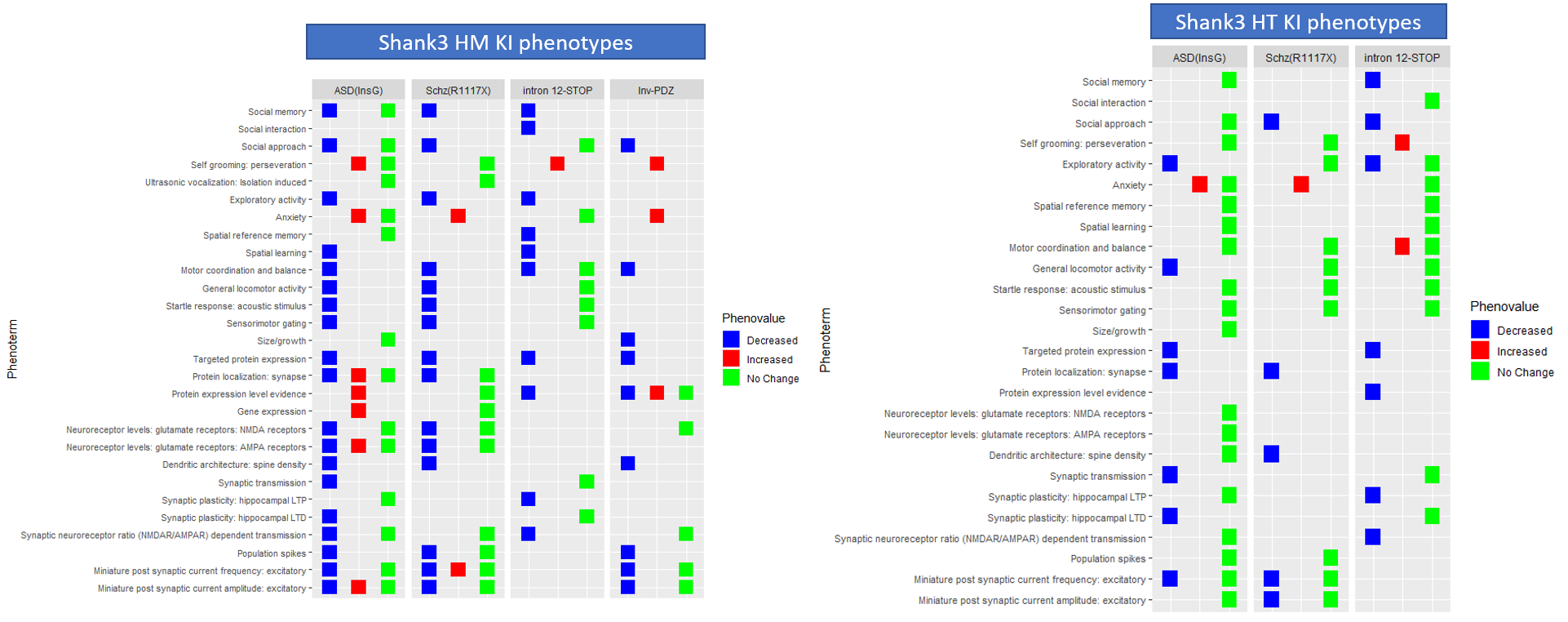

Supplement: Supplementary file 7 — Figure S5. Phenotypes of Shank3 KI models. A) The HM KI models display several core phenotypes including impaired social behavior and increased self-grooming. Depending on the mutation, there is heterogeneity in the manifestation of other behavioral phenotypes like anxiety and spatial learning. The KI human mutations are discussed in main text. B) Shank3 HT KI mutant mice still manifest core phenotypes, whereas other tested behavior is more like wild-type mice, like normal anxiety, sensorimotor gating, and spatial learning. (TIF 707 kb) [file 13229_2019_263_MOESM7_ESM.tif]

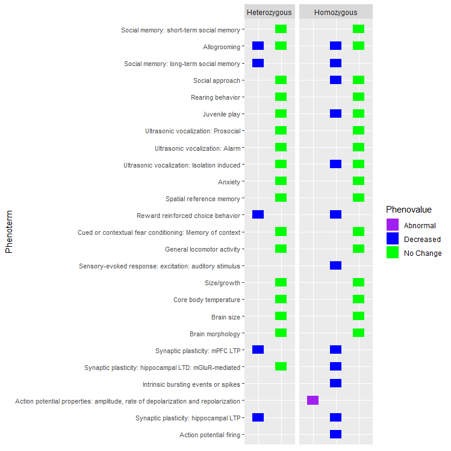

Supplement: Supplementary file 8 — Figure S6. Rat Shank3 phenotypic data. The HT and HM rat models depicted here were developed by targeting the Ank domain. Rat models of Shank3 display some deficits in social behavior (long-term memory) but do not share several of the phenotypes displayed by mouse Shank3 models, like no impairments in ultrasonic vocalization or changes in anxiety levels are observed in rats. (TIF 105 kb) [file 13229_2019_263_MOESM8_ESM.tif]
